# A systematic review of help-seeking interventions for depression, anxiety and general psychological distress

**DOI:** 10.1186/1471-244X-12-81

**Published:** 2012-07-16

**Authors:** Amelia Gulliver, Kathleen M Griffiths, Helen Christensen, Jacqueline L Brewer

**Affiliations:** 1 Centre for Mental Health Research, The Australian National University, Canberra, Australia

## Abstract

**Background:**

Depression and anxiety are treatable disorders, yet many people do not seek professional help. Interventions designed to improve help-seeking attitudes and increase help-seeking intentions and behaviour have been evaluated in recent times. However, there have been no systematic reviews of the efficacy or effectiveness of these interventions in promoting help-seeking. Therefore, this paper reports a systematic review of published randomised controlled trials targeting help-seeking attitudes, intentions or behaviours for depression, anxiety, and general psychological distress.

**Methods:**

Studies were identified through searches of PubMed, PsycInfo, and the Cochrane database in November 2011. Studies were included if they included a randomised controlled trial of at least one intervention targeting help-seeking for depression or anxiety or general psychological distress, and contained extractable data on help-seeking attitudes or intentions or behaviour. Studies were excluded if they focused on problems or conditions other than the target (e.g., substance use, eating disorder).

**Results:**

Six published studies of randomised controlled trials investigating eight different interventions for help-seeking were identified. The majority of trials targeted young adults. Mental health literacy content was effective (*d* = .12 to .53) in improving help-seeking attitudes in the majority of studies at post-intervention, but had no effect on help-seeking behaviour (*d* = −.01, .02). There was less evidence for other intervention types such as efforts to destigmatise or provide help-seeking source information.

**Conclusions:**

Mental health literacy interventions are a promising method for promoting positive help-seeking attitudes, but there is no evidence that it leads to help-seeking behaviour. Further research investigating the effects of interventions on attitudes, intentions, and behaviour is required.

## Background

Mental disorders are a leading cause of years lost due to disability and significantly contribute to the global burden of disease [1]. Common mental disorders such as depression and anxiety disorders are treatable, and potentially preventable [2-4]. Appropriate help-seeking from a professional source is therefore particularly important for the prevention, early detection and treatment of, and recovery from mental disorders [5,6]. Despite this, it is estimated that only one quarter of adults with high levels of mental distress [7] and one third of adults with diagnosable mental disorders seek professional help [8]. Given the debilitating nature of depression and anxiety disorders and the existence of effective treatments, there is increasing recognition of the importance of promoting appropriate help-seeking for mental health problems in these groups.

### Prevalence and burden

Depression and anxiety are highly prevalent mental disorders with an estimated 12-month prevalence of 14-18% for anxiety disorders, and 4-7% for depressive disorders in high income countries worldwide [9-11]. In addition, almost 33% of the Australian population suffers from at least moderate general psychological distress, with 12% of those suffering high to very high distress in the 12-months prior to the survey [11]. Depression, anxiety and general psychological distress contribute significantly to societal disease burden, including the economic impact of lowered work productivity, and frequent use of medical services [8,12]. These conditions also can have severe consequences for the individual if left untreated including disability [8,13], suicide [14], lowered quality of life, and physical and social functioning, even for those experiencing sub-clinical symptoms of depression or anxiety [15].

### Help-seeking for anxiety and depression

Despite the severity of these consequences, it is estimated that up to one-half of those with depression [16-18] and only one third to one-half of those affected by anxiety disorders [8,16] seek professional help. Moreover, people often seek help from informal sources, such as friends or family rather than from formal sources such as doctors or psychologists [19,20] who can provide evidence-based treatments [21]. There is therefore a clear need to promote greater help-seeking from evidence-based sources.

### Help-seeking theory

Several theories and models have been applied to help-seeking for mental health problems but none has been widely accepted. Azjen’s theory of planned behavior [22] is concerned with how attitudes, subjective norms, and perceived control over the behaviour interact to influence intentions, and consequently the behaviour itself. Recently, this theory has been used to demonstrate the mediating effect of attitudes on men’s psychological help-seeking intentions [23]. Another approach, the health belief model, posits that the decision to perform a behaviour is dependent on the individual’s appraisal of the perceived threat of illness and its severity, and the perceived barriers and benefits of the behaviour itself [24]. The health belief model has previously been utilised as a framework for understanding help-seeking behaviour in the general population for mental health problems [25]. Finally, Andersen’s behavioral model [26,27] describes a 3-stage model for health services use describing the factors of predisposing characteristics such as the individual’s demographic information and beliefs, enabling resources such as cost and access to care, and illness level which is interpreted as the individual’s perceived and evaluated need for help. This model has been applied to treatment-seeking for panic attacks in community-based adults [28] and help-seeking for mental health problems in refugees [29].

There are also several models that were developed specifically for mental health help-seeking. The first approach conceptualising help-seeking involves a dynamic model that focuses on why young people do not seek help [30]. This model describes non-help-seeking in terms of a circular process, *the cycle of avoidance*, which is influenced by the three interacting factors including the individual’s conceptualisation of mental distress, what they believe it means in society to seek help, and their own purposeful action of seeking help. The final model [31], which is also concerned with help-seeking among young people, conceptualises seeking professional help as a multi-step process beginning with the individual’s development of an awareness of the problem, followed by the expression of the problem and a need for help to others, the identification of appropriate of sources of help available for the individual to access, and finally, the willingness of the individual to actually seek out and disclose to potential sources of help.

### Types of help-seeking interventions

None of the above models or theories is universally agreed upon in the help-seeking field. Despite this lack of a common framework, research investigating the nature and extent of help-seeking has tended to examine three broad aspects of help-seeking: *attitudes* towards help-seeking including beliefs or willingness to seek help, help-seeking *intentions*, and actual help-seeking *behaviour*. Since there is evidence that attitudes and intentions can predict behaviour [32], there is a possibility that improving these two aspects could be beneficial, in addition to targeting help-seeking behaviour. Accordingly, much of the intervention research on help-seeking has focused on improving help-seeking attitudes and intentions, with the aim of producing behavioural change, as well as targeting the behaviour itself.

Consistent with the broad theoretical frameworks outlined above, the interventions have incorporated a range of content types, often in combination. These include *mental health literacy* defined as “knowledge and beliefs about mental disorders which aid their recognition, management or prevention” [33], *destigmatisation*, which refers to the reduction of stigma surrounding mental disorders [34] often involving personal *contact with consumers* of mental health services [35], providing *help-seeking source information* about how and where to find potential providers of help [36], *contact* with the researchers or interviewers*,* and less commonly, *cognitive behavioural therapy (CBT)* and *personalised feedback*[37] about the individual’s symptoms with the aim of prompting help-seeking in those with higher symptom levels.

Help-seeking intervention research has also used two main approaches; universal interventions aimed at the general population, and indicated interventions targeted at those who have mild or early symptoms of a disorder [38].

### Aims and scope of study

Despite the importance of increasing help-seeking for mental health problems, to date there have been no systematic reviews of the efficacy or effectiveness of interventions designed to promote help-seeking. Therefore, this study aims to systematically review randomised controlled trials of the efficacy or effectiveness of interventions designed to increase professional help-seeking for depression, anxiety and general psychological distress. The review conforms to the PRISMA statement [39]. A PRISMA checklist is provided in Additional file 1: PRISMA 2009 Checklist.

## Methods

### Search methodology and inclusion criteria

The Cochrane Library, PubMed, and PsycINFO databases were searched in November 2011 using the search terms presented in Additional file 2: Search terms. These terms aimed to represent the primary concepts of ‘help-seeking’, and ‘mental health’ or ‘mental disorders’ in a randomised controlled trial. Keywords were combined with MeSH terms from the PubMed and Cochrane databases and Subject Headings for the PsycINFO database, and were restricted through the limit function for all databases to ‘trial’ papers.

**Figure 1:**
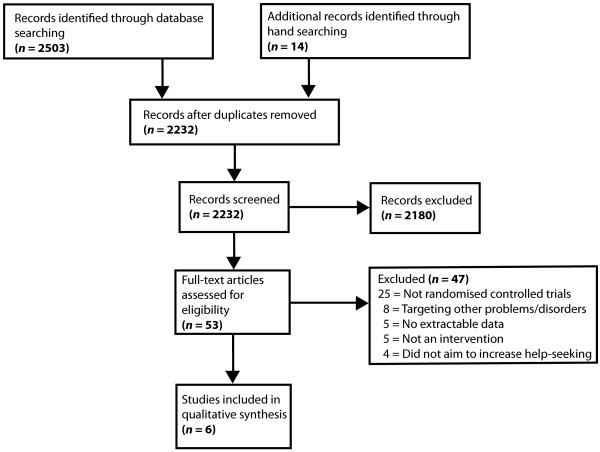
Study selection flow diagram.

Figure 1 presents the flow chart for the selection of the included studies. After removing duplicates, the database search yielded 2232 published English-language abstracts. One of the researchers (AG) subsequently screened the titles and abstracts for relevant papers, which resulted in 39 potentially relevant studies. An additional 14 studies were located through hand-searching the reference lists of key papers found through the systematic search and which were considered likely to satisfy the inclusion criteria. This yielded a total of 53 papers for possible inclusion in the review.

The inclusion criteria for the present review were that the study a) was a randomised controlled trial of at least one intervention, b) contained extractable data on the help-seeking outcomes of attitudes or intentions or behaviour, and c) employed an intervention/s designed to improve help-seeking attitudes or increase help-seeking intentions or behaviour for depression, anxiety or general psychological distress. Studies focused on problems or conditions other than the target conditions (e.g., substance use, eating disorder, occupational trauma risk management, or suicide prevention) were excluded. A total of 47 studies did not meet the inclusion criteria and were excluded from further consideration. Excluded studies are listed in Additional file 3: List of studies excluded from the review by exclusion category. No study was excluded on the basis of research quality. This left a total of six relevant studies [37,40-44].

### Data extraction

Included studies were each coded with a pre-formulated rating sheet with relevant data extracted and recorded. Data coded included author, location of study, age range and number of participants, intervention comparisons, delivery mode, intervention provider, setting and length of the intervention/s, help-seeking measures, the results of the study, and quality ratings. Coding was carried out independently by two of the study authors (AG, JB) and coding discrepancies were resolved by discussion between the coders. Only data on help-seeking from professional sources was coded (i.e., general practitioner or a mental health professional). If the required data was not available, the author was contacted via email to request information. A formal meta-analysis was not conducted because the outcome measures were diverse.

### Effect size calculations

In order to compare the relative efficacy or effectiveness of the studies, between group effect sizes were estimated using an effect size calculator [45] to calculate Cohen’s *d*[46] at post-test and follow-up where provided. A positive effect size denotes a greater improvement in scores in the intervention group than the control group at post-test or follow-up.

### Quality ratings

The study quality was assessed using the nine-item EPOC risk of bias tool [47], a measure designed to assess potential sources of bias for studies involving a control group. Items are designed to measure bias relating to inadequate random allocation sequence and allocation concealment, differences in baseline outcome measurements and characteristics, inadequate treatment of missing outcome data, researcher knowledge of allocated interventions, contamination between the conditions, and selective outcome reporting as well as any other risk of bias. A score of 1 was awarded for each criterion adequately addressed within the paper with potential scores ranging from 0 to 9.

## Results

### Study characteristics

The characteristics, efficacy or effectiveness data, and quality ratings of the six studies are presented in Table 1.

**Table 1:** **Randomised controlled trials included in the review (*n*** **= 6)**

**#**	**Author**	**Loc**	**Age**	***N***	***N***	**Intervention comparisons**	**Delivery mode**	**Provider**	**Setting & recruitment**	**Length**	**Help-seeking measures**	**Post-test effect size**	**Follow-up effect size**	**Quality rating**
				**Randomised**	**Post, FU**									
1	Christensen	AUS	18-52	*N* = 525	Post *N* = 414	Two web-based depression interventions with weekly telephone calls from interviewer (indicated for participants K-10 ≥12) vs. control:	Online (Web plus telephone)	Interviewer	Community survey sent to random selection of 27,000 people on the Australian electoral roll (compulsory registration), Response rate: 6130 (22.7%);	6 wks	(1) Behaviour – Self-reported professional treatments sought in the past 2 months to cope with depression *(includes GP, counsellors, psychologists, anti-depressants, CBT, self-help books)*	6 weeks:	6 months:	6/9
	(2006) [37]											(1) **MG** = .24^a^	(1) **MG** = .13	
			*M* = 36.8	**BP** = 165	**BP** = 136							(1) **BP** = -.01	(1) **BP** = .02	(3, 4, 5)
			*SD* = 9.3	**MG** = 182	**MG** = 121									
				**C** = 178	**C** = 157									
					FU *N* = 346	**BP** = BluePages (MHL for depression + feedback).								
					**BP** = 114	**MG** = MoodGYM (CBT program for depression + feedback).			657 (2.4%) met inclusion criteria.					
					**MG** = 102									
					**C** = 130									
						**C** = Control (weekly telephone calls from interviewer only about lifestyle factors that may influence depression).								
2	Costin (2009)	AUS	19-24	*N* = 348	*N* = 298	Two e-card interventions vs. control:	Online (Email)	Researcher	Community survey sent to 12,000 young people aged 19-23 years on the Australian electoral roll, (compulsory registration), Response rate: 1764 (14.7%); 1189 (9.9%) met inclusion criteria.	3 wks	(1) Behaviour – sought help in the past 6 wks from formal sources (AHSQ)	6 weeks:	*NR*	9/9
	[40]											**BS** and **EH** combined by author -		
			*M* = 21.4	**BS** = 114	**BS** = 97									
			*SD* = 1.5	**EH** = 117	**EH** = 97	**BS** = Basic (basic MHL for depression and help-seeking sources information).								
				**C** = 117	**C** = 104							(1) **BS**/**EH** = -.02		
											(2) Intentions – to seek help from formal sources (GHSQ)	(2) **BS**/**EH** = .03		
						**EH** = Enhanced (enhanced MHL for depression and help-seeking sources information).						(3) **BS**/**EH** = .53^a^		
						**C** = Control (general health issue information).					(3) Beliefs ^e^ – rated any formal source as helpful			
3	Buckley	AUS	18-79	*N* = 80	Post *N* = 80	One 30 minute video vs. control:	In-person (Video)	Assistants (not directly involved in study) ^d^	University student volunteers (30) and community members (50) volunteering from specified groups (e.g., teachers, church) ^d^	30 mins	(1) Attitudes (ATSPPHS)	30 minutes:	2 weeks:	5/9
	(2005) [44]											(1) **VID** = .34^b^	(1) **VID** = .56^b^	
			*M* = 40.6	**VID** = 39	**VID** = 39									(1, 2, 4, 9)
			*SD* = 19.2	**C** = 41	**C** = 41	**VID** = Video [based on cognitive learning theory, first person accounts of psychotherapy experiences (consumer contact), MHL, destigmatisation].								
					FU *N* = 63									
					**VID** = 29									
					**C** = 34									
						**C** = Control (30 minute video of “the self” with no mental health treatment mentioned).								
4	Donohue	USA	17-49 ^d^	*N* = 124	Post *N* = 124	One interview vs. control:	In-person	Research assistant	University student athletes recruited via university notices. The majority received psychology course credit for participation.	10-15 mins	(1) Attitudes	10-15 minutes:	*NR*	6/9
	(2004) [41]						(Interview)				(ATSSPCQ)	(1) **INT** = .12^b^		
			*M* = 19.6	**INT** = 60 ^d^	**INT** = 60 ^d^	**INT** = Interview [discussing sport psychology and its benefits to the athlete (MHL, help-seeking source information)].						(2) **INT** = .34		(1, 2, 5)
			*SD* = 1.8	**C**= 64 ^d^	**C**= 64 ^d^						(1) Attitudes: Confidence in sport psychology consultation	(3) **INT** = -.08		
						**C** = Control (interview discussing general experiences in sport).					(2) Attitudes: Personal openness			
											(3) Attitudes: Stigma tolerance			
5	Han (2006)	TAI	18-36	*N* = 299	Post *N* = 243	Three written material interventions vs. control:	In-person (Written)	Researcher^d^	University students drawn from student body of 3 universities with 144 receiving psychology course credit for participation.	5-10 mins	(1) Willingness^e^ (HSWS)	2 weeks:	*NR*	5/9
	[42]											**BA** and **DS** Combined across conditions for results. However, individual group effect sizes were:		
			*M* = 20.3	**BA** = 75	**BA** = 64									(1, 2, 4, 5)
			*SD* = 2.2	**DS** = 76	**DS** = 56	**BA** = Biological attribution of depression psychoeducation (MHL).								
				**CM** = 72	**CM** = 61									
				**C** = 76	**C** = 62									
						**DS** = Destigmatisation of depression.								
												(1) **BA** = .17^a c^		
						**CM** = Combined BA and DS.						(1) **DS** = .04^c^		
						**C** = Control (no information).						(1) **CM** = .32^a c^		
6	Sharp (2006)	USA	18-43	*N* = 123	Post *N* = 115	One seminar vs. control:	In-person (Seminar plus written)	Clinical psychology graduate student with master’s degree	University students seeking to fulfil psychology course requirement.	40 mins	(1) Attitudes (ATSPPHS-SF)	1 week:	4 weeks:	6/9
	[43]											(1) **SEM** = .26^b^	(1) **SEM** = .26^b^	
			*M* = 20.0	**SEM** = 62	**SEM** = 60	**SEM** = Seminar [classroom-based mental health psychoeducational seminar + written information handouts (MHL, destigmatisation, help-seeking source information].					(2) Behaviour (Self-report help-seeking from mental health professional in the last 4 wks).	(2) **SEM** = *NR*	(2) **SEM** = .01	(1, 2, 4)
			*SD* = 3.2	**C** = 61	**C** = 55									
					FU *N* = 105									
					**SEM** = 57									
					**C** = 48	**C** = Control (astronomy science video.								

**Note:** All studies were randomised at the individual level. **Author** = First author; **Loc** = Location of study, AUS = Australia, USA = United States of America, TAI = Taiwan; **Age** = Age range if provided, M = mean, SD = standard deviation of participants’ age; ***N*Randomised** = total number of participants randomised; ***N*Post, FU** = Number at post-intervention and follow-up; **Intervention comparisons** = Description of interventions; **Delivery mode** = Delivery mode; **Provider** = Person providing or facilitating the intervention; **Setting =** Where was the study recruited from and conducted; **Length** = Length of intervention; **Help-seeking measures** = Measures of professional help-seeking used; **Post-test effect size/Follow-up effect size** = Effect size calculated using Cohen’s d participant distributions (nominal data), or means and standard deviations using the Practical Meta-Analysis Effect Size Calculator [45]; **Quality rating** = Quality rating of study using EPOC criteria - Numbers included in column to indicate which criteria study did not adequately address and report; **1** = Was the allocation sequence adequately generated?; **2** = Was the allocation adequately concealed?; **3** = Were baseline outcome measurements similar?; **4** = Were baseline characteristics similar?; **5** = Were incomplete outcome data adequately addressed?; **6** = Was knowledge of the allocated interventions adequately prevented during the study?; **7** = Was the study adequately protected against contamination?; **8** = Was the study free from selective outcome reporting?; **9** = Was the study free from other risks of bias?; Means and standard deviations were rounded to one decimal place, effect sizes to two decimal places.

^a^ Significant difference between intervention and control groups at post-test.

^b^ Significant difference between intervention and control groups in change scores from pre- to post-test.

^c^ Results combined by authors across conditions.

^d^ Sourced information from author post-publication.

^e^ Categorised as “attitudes” based on items from the scale.

### Year and location of studies

The studies were published between 2004 and 2009 with three conducted in Australia [37,40,44], two in the United States [41,43], and one in Taiwan [42].

### Sample and participant characteristics

#### Sample size

Participant numbers ranged from 80 to 525.

#### Participant age and target population

The range of ages was 17 to 79 years across the studies. Four of the studies [40-43] were conducted with young people with a mean age of less than 25 years and one of these [40] specifically targeted young people aged 19 to 24 years. No studies targeted school-aged children. Five out of the six trials involved universal interventions [40-44]. One trial involved an indicated intervention [37] and included participants with high levels of psychological distress only (K-10 scores above or equal to 12).

#### Setting and delivery mode

Three of the studies were conducted in universities and targeted undergraduate students [41-43], two recruited participants from the general community [37,40], and one was recruited from two different locations; a university and a church complex in the community [44]. Four of the studies were conducted in-person [41-44], each employing a different delivery mode (video, interview, written information, seminar plus written information). Two studies were conducted online, one via email [40], and one via internet websites plus telephone contact with interviewers [37].

#### Providers and intervention length

Provider type varied. In three studies the intervention was delivered by the researchers themselves or a research assistant [40-42], two studies used an interviewer or an assistant [37,44], and one study employed a clinical psychology graduate with a master’s degree [43]. The four in-person studies were very brief and ranged from 5–10 minutes to 40 minutes in total duration over one session [41-44]. The two online interventions were delivered over 3 and 6 weeks for the email and internet websites plus telephone interventions respectively [37,40].

### Evaluation measures and intervention content

#### Help-seeking measures

None of the measures was the same across studies. Four of the six studies used self-report measures of attitudes to or beliefs about professional help-seeking [40,41,43,44], three studies measured help-seeking behaviour from a professional source [37,40,43], one study measured intentions to seek help from a professional source [40], and one study measured willingness to seek help from a professional source [42].

#### Intervention content

There were ten intervention conditions of varying content and six control conditions, of which five employed an attention placebo [37,40,41,43,44] and one involved no intervention [42]. The author of one study combined the results of two intervention conditions prior to analysis [40], and the authors of another split the components of one combined condition in a factorial design for analysis [42]. This left a total of eight intervention conditions for analysis. Most of these intervention conditions involved several components. Therefore, studies were categorised under each component and the total for the following analysis does not equal the total number of interventions. The majority (*n* = 6) included a component of information targeting mental health literacy as part of their content [37,40-44]. Other approaches included destigmatisation information (*n* = 3) [42-44], providing help-seeking source information (*n* = 3) [40,41,43], weekly interviewer contact via telephone (*n* = 2) [37], contact with consumers in the form of first person accounts of psychotherapy via a pre-recorded video (*n* = 1) [44], online CBT using cognitive restructuring, behavioural techniques such as pleasant events scheduling, relaxation, and problem solving (*n* = 1) [37], and personalised feedback about the individual’s symptoms (*n* = 2) [37].

### Trial outcomes

#### Overall findings

For all six trials [37,40-44], there was a significant improvement at post-test on at least one help-seeking measure for at least one intervention condition compared with the control group. Specifically, all of the trials measuring attitudes, willingness, or beliefs (*n* = 5) [40-44] found a significant improvement in help-seeking attitudes at post-test for at least one of their intervention conditions compared with the control, with effect sizes ranging from .12 to .53. However, only one of three studies measuring behaviour found a significant improvement in help-seeking behaviour at post-test and this effect size was relatively small (*d* = .24) [37]. In this study, the successful intervention used CBT and personalised feedback, whilst the unsuccessful intervention used information targeting mental health and personalised feedback. Finally, there was no significant intervention effect in the only study to measure help-seeking intentions at post-test [40]. The median effect size at post-test was .17 (range = −.08 to.53).

Half of the studies [37,43,44] provided follow-up data either at 2 weeks, 4 weeks, or 6 months post-test, and of these the two with the shortest follow-up periods found a significant improvement in help-seeking attitudes (2 weeks, *d* = .56; 4 weeks, *d* = .26) [43,44].

#### Intervention type

Six of the eight interventions delivered information targeting *mental health literacy*. Of these, five [40-44] measured and reported a significant improvement in help-seeking attitudes at post-test compared with the control. Help-seeking behaviour was measured for three interventions [37,40,43], but no differences between the control and experimental condition were found for help-seeking behaviour at post-test for the interventions delivering information targeting mental health literacy.

For two of the three interventions that delivered *destigmatisation* information, help-seeking attitudes improved [43,44], while for one it did not [42]. The one study that also measured help-seeking behaviour [43] did not find significantly greater help-seeking behaviour among the intervention compared with the control group at post-test.

All three of the interventions delivering *help-seeking source information*[40,41,43], reported a significant improvement in help-seeking attitudes among the intervention group compared with the control at post-test. Of these, one measured behaviour [43] and found no significant effect of the intervention on this aspect of help-seeking. Of the two interventions within the same trial using *interviewer contact via telephone*[37], one demonstrated a significant increase in help-seeking behaviour in the intervention group when compared with the control, and one did not. The intervention that provided *contact with consumers* (via a pre-recorded video) [44] was associated with a significant improvement in attitudes compared with the control. Similarly, the intervention that provided *CBT*, and *personalised feedback* about the individual’s symptoms [37] significantly improved help-seeking behaviour in the comparison group compared with the control.

#### Study quality

The study quality scores ranged from 5 to 9, with one study with one study receiving a score of 9 [40], three studies a score of six [37,41,43], and two a score of five [42,44].

## Discussion

The present review identified six randomised controlled trials of help-seeking interventions for depression, anxiety, and general psychological distress. Overall the results indicated that improvement in some aspects of help-seeking was achievable, with all of the studies finding a positive effect relative to control for at least one intervention on at least one aspect of help-seeking attitudes, intentions or behaviour. However, median effect sizes were small.

It is difficult to locate reliable trends in the data for intervention content with so few studies. However, almost all of the interventions that delivered information targeting mental health literacy were associated with improved attitudes, although none of them were able to influence help-seeking behaviour out of the three studies measuring this aspect. Results were mixed for the interventions delivering destigmatisation information, with two out of the three finding significantly improved attitudes at post-test [43,44]. Providing information about help-seeking sources was common to three interventions [40,41,43], and all three successfully improved attitudes. Finally, there were no discernable effects for contact with mental health consumers [44], or contact with the research team via telephone [37]. The only study to produce reported behaviour change was that involving CBT and personalised feedback about symptoms.

Importantly, in almost all studies the interventions incorporated more than one type of content. This made assessing the individual contributions of content components problematic [48]. However, one study did attempt to dismantle the effects of the content of messages on help-seeking willingness for depression [42]. They compared the effect of providing information about the physiological aspects of depression including genes, neurotransmitters, and endocrine systems (biological attribution condition) with providing information aimed at reducing psychological blameworthy attitudes towards depression. They concluded that only the biological attribution intervention increased willingness to seek help. This is an important result, as it is possible that less content may be needed to facilitate help-seeking than is currently being trialled. Further research in this area is required to assess the efficacy of individual content components with a view to ensuring future interventions are comprised of brief but highly effective components [48].

It is encouraging that all studies found a positive outcome on at least one measure of help-seeking. It appears that attitudes, including the measures of ‘beliefs’ and ‘willingness’, may be the most malleable of the three help-seeking facets, as all of the trials measuring this outcome found a significant improvement at post-test for at least one of their comparison conditions [40-44]. Help-seeking *intentions* and *behaviour* may be more difficult to change, with the only study of help-seeking *intentions*[40] and two of the three studies measuring help-seeking *behaviour* failing to find a positive effect compared to the control [37]. Theories of help-seeking predict that attitudes and beliefs influence behaviour [22,24,26,27,31]. However, currently there is little empirical evidence to support this hypothesis with respect to mental illness. In fact, it is known that attitudes do not necessarily translate into behaviour [42]. In the case of help-seeking for mental illness, behaviour may be more difficult to change than beliefs as it is external and thus potentially more vulnerable to the stigmatising of others, which in turn has been linked to help-seeking avoidance [49]. Further work is needed to develop interventions that are effective agents for help-seeking behaviour change.

Three of the studies provided follow-up data at 2 weeks, 4 weeks and 6 months respectively. Both studies that measured the shorter follow-up time period [43,44] measured attitudes and reported a significant positive effect of the intervention at both post-test and follow-up but the study with the longest follow-up time measuring behaviour found an effect at immediate post-test only [37]. This raises questions about the sustainability of the effects of the interventions in general as well as on the different aspects of help-seeking. Thus, further studies investigating the longer term effects of help-seeking interventions are required.

Study quality was moderate, with all of the studies successfully satisfying at least five of the nine quality criteria. However, there was poor adherence to four of the criteria. In particular, a number of studies failed to describe or adequately generate the allocation sequence for randomisation, conceal the unit of allocation, provide the baseline characteristics of providers, or address incomplete outcome data. It is important for future trial research to appropriately address and report on these potential areas of bias.

Almost all of the studies used an attention placebo rather than a less conservative control such as a Wait List or no intervention Control. Thus, it is unlikely that the positive effects that were observed on help-seeking attitudes and behaviour were the consequence of non-specific factors such as social support or attention [48]. Future help-seeking intervention research should continue to utilise attention placebos, particularly those in which the content is unlikely to affect the primary outcomes.

The results of the present review demonstrate that the majority of randomised controlled trials investigating help-seeking for common mental health problems and general psychological distress target young people. A focus on this age group is appropriate given that the prevalence of mental disorders is highest in adolescents and young adults [11]. However, there is also a need for further research involving other age groups.

Additionally, almost all of the present study trials were conducted with universal samples reflecting that the current focus in the literature is on promoting positive help-seeking attitudes and intentions prior to the development of symptoms. Detecting help-seeking change in universal populations employing short-term follow-up periods may be difficult given that only a minority of the target group will have a mental disorder that requires help. It would be expected that help-seeking behaviour would be more likely if the individual is symptomatic and therefore had a perceived need to seek help [26,27]. Consistent with this, the single trial [37] that did investigate the effects of the intervention on a symptomatic population found positive results for behaviour. Given the importance of help-seeking behaviour for those experiencing current symptoms [21], this is an essential area for future research.

The majority of trials were conducted in person. However, two of the more recent trials involved content delivered via the internet [37,40] and both successfully increased professional help-seeking behaviour or intentions. There is an increasing focus on delivering mental health prevention and treatment services over the internet, with research indicating that online services are highly acceptable to young people [50]. Additionally, a survey of over 50,000 young people in Australia [51] indicated that after parents, relatives and friends, the next most common source of advice and support for personal problems is the internet. It has been reported that the online delivery of interventions targeting health-related behavioural change are as effective as face-to-face forms of delivery [52]. In addition, online delivery may be a relatively inexpensive means of delivering treatments to a wide range of people [53], particularly for rural residents [54] whose access to in-person interventions may be limited. Given the reported positive effects of the internet interventions in the present study on formal help-seeking, there may be value in using online help-seeking applications to promote the use of online evidence-based treatment services. Further research is required to explore this possibility and the cost-effectiveness of such models.

With respect to length, the duration of the interventions varied from 5–10 minutes to 6 weeks. The longest, and possibly the most intensive intervention utilising CBT and brief feedback for depression [37], was the only one that successfully increased professional help-seeking behaviour, out of three studies measuring behaviour [37,40,43]. However, this study was also the only trial to specifically select participants with psychological symptoms, and thus a greater proportion of this than the other trial groups had a need for professional help [55,56].

All of the measures of help-seeking involved self-report, although they varied in type and in what they measured. The studies in the present review rarely measured attitudes, intentions, and behaviour in the one study. Given that these three may be impacted on differently by different interventions, and indeed different components of the interventions, it may be important to measure all of these outcomes in the same trial. However, this might require long-term follow-up to detect effects on behaviour if the studies involve universal populations unselected for symptom levels. In addition, there is a need for consensus on the most appropriate measures of help-seeking to facilitate comparison between studies. Further, it may be useful to assess knowledge about and stigma towards help-seeking for mental disorders in order to understand more about the help-seeking process. Providing destigmatising material as well as measuring its effect is particularly important, as subjective norms or beliefs about what others think about help-seeking, are thought to influence intentions [22]. Research to refine and test models of help-seeking for mental disorders is warranted. In addition, only one study [40] in the present research designed an intervention based on a help-seeking model. It may be of benefit to the help-seeking field if future researchers were to use a model or theory of help-seeking as a basis for their intervention design. As Costin et al. [40] noted with respect to the Rickwood et al. model [31], this may allow the targeting of model-specific factors that could inhibit the progression through the help-seeking process, such as how to contact a mental health professional, and what to expect in a consultation.

### Limitations

There are some limitations to the present review that require consideration. Firstly, the measures of help-seeking found varied and this lack of standardisation makes comparison between studies difficult. Secondly, only one study investigated actual help-seeking behaviour; it is possible that positive help-seeking attitudes and intentions do not translate into action.

The present review searched three databases and it is possible that some relevant journals are not indexed by these databases. However, an attempt was made to address this by hand-searching the reference lists of papers that were captured [57]. In addition, the restriction of the inclusion criteria to English-language journal papers may have introduced a level of bias into the present review, as may the incorporation of published papers only given that publication may be biased towards papers with positive outcomes [58].

A final limitation is the subjective nature of the coding. To address this, two coders extracted data from each paper and discrepancies were discussed and resolved.

## Conclusions

Overall, the present findings provide some evidence that mental health literacy help-seeking interventions targeting depression, anxiety, and general psychological distress can be effective in increasing help-seeking *attitudes*. Significantly however, very little is known about what interventions increase help-seeking *behaviour*. Further research underpinned by models of help-seeking which facilitate the design of interventions are required if we are to address the substantial public health problems associated with the common mental disorders of depression and anxiety.

## Competing interests

Kathleen Griffiths and Helen Christensen are co-developers of MoodGYM and Bluepages, which were both evaluated as part of one of the included trials [37]. Additionally, both were co-authors of another included trial [40].

## Authors’ contributions

AG designed the study and the search criteria, developed coding checklists, undertook the analyses, coded the papers and wrote a draft of the manuscript. KG and HC supervised all stages of the research, contributed to the design and analysis of the study and critically edited the paper. JB assisted with the development of coding checklists, coded the papers and provided comments on the paper. All authors read and approved the final manuscript.

## Supplementary Material

Additional file 1 Microsoft word document. PRISMA 2009 Checklist. Click here for file

Additional file 2 Microsoft word document. Search terms. Click here for file

Additional file 3 Microsoft word document. List of studies excluded from the review by exclusion category. Click here for file
