# Supplementary material for: A systematic review of help-seeking interventions for depression, anxiety and general psychological distress
Source: BMC Psychiatry. 2012 Jul 16;12:81. doi: 10.1186/1471-244X-12-81 (PMC3464688; doi:10.1186/1471-244X-12-81)
Supplement: Additional file 2 — Microsoft word document. Search terms. [file 1471-244X-12-81-S2.doc]

**Search Terms**

***SEARCH 1: PsycINFO – (conducted 08/11/11 =1365)***

1. exp Mental Health/

2. exp Mental Disorders/

3. 1 or 2

4. (Helpseek$ or Seek$ help or Seek$ treatment).mp. [mp=title, abstract, heading word, table of contents, key concepts]

5. exp Help Seeking Behavior/

6. 4 or 5

7. 3 and 6

8. limit 7 to (human and english language and "0400 empirical study" and (200 adolescence <age 13 to 17 yrs> or "300 adulthood <age 18 yrs and older>") and "0100 journal")

***SEARCH 2: PubMed (conducted 08/11/11 =769)***

1. Mental Health or Mental disorder [MeSH]

2. Help seek* or Seek* help or Seek* treatment

3. 1 and 2

4. limit 3 to (Humans, Clinical Trial, Randomized Controlled Trial, Controlled Clinical Trial, English, All Adult: 19+ years, Adolescent: 13-18 years)

***SEARCH 3: Cochrane (conducted 08/11/11 =369)***

1. Mental Health or depress* or anxiety

2. Help seek* or Seek* help or Seek* treatment

3. 1 and 2

4. limit 3 to [Cochrane Central Register of Controlled Trials (Clinical Trials)]
