# Supplementary material for: A systematic review of help-seeking interventions for depression, anxiety and general psychological distress
Source: BMC Psychiatry. 2012 Jul 16;12:81. doi: 10.1186/1471-244X-12-81 (PMC3464688; doi:10.1186/1471-244X-12-81)
Supplement: Additional file 3 — Microsoft word document. List of studies excluded from the review by exclusion category. [file 1471-244X-12-81-S3.doc]

**List of studies excluded from the review by exclusion category**

Studies were excluded because they: (1) were not randomised controlled trials (*n* = 25; before and after study = 11, controlled trial = 9, randomised trial = 5), (2) targeted other disorders or problems (*n* = 8; substance use = 3, suicide prevention = 2, occupational trauma risk management = 2, eating disorder=1), (3) did not contain extractable data (*n* = 5), (4) were not an intervention (*n* = 5), or (5) did not aim to increase help-seeking (*n* = 4).

The following list details these excluded studies according to the primary reason for their exclusion.

**Criterion 1. Not randomised controlled trials (*n* = 25)**

*Before and after study (n = 11)*

| 1 | Barker, C., Pistrang, N., Shapiro, D. A., Davies, S., & et al. (1993). You in Mind: A preventive mental health television series. *British Journal of Clinical Psychology, 32*(3), 281-293. |
| --- | --- |
| 2 | Burns, J. M., Durkin, L. A., & Nicholas, J. (2009). Mental health of young people in the United States: What role can the Internet play in reducing stigma and promoting help seeking? *Journal of Adolescent Health, 45*(1), 95-97. |
| 3 | Chovil, l., & Geller, J. L. (2004). Help-Seeking Preferences of High School Students: The Impact of Personal Narratives. *Psychiatric Services, 55*(8), 863-865. |
| 4 | Nicholas, J., Oliver, K., Lee, K., & O'Brien, M. (2004). Help-seeking behaviour and the Internet: An investigation among Australian adolescents. *Australian eJournal for the Advancement of Mental Health, 3*(1), 1-8. |
| 5 | Bhugra, D., & Hicks, M. H. (2004). Effect of an educational pamphlet on help-seeking attitudes for depression among British South Asian women. *Psychiatr Serv, 55*(7), 827-829. |
| 6 | Greenfield, S. F., Reizes, J. M., Magruder, K. M., Muenz, L. R., Kopans, B., & Jacobs, D. G. (1997). Effectiveness of community-based screening for depression. *Am J Psychiatry, 154*(10), 1391-1397. |
| 7 | Teng, E. J., & Friedman, L. C. (2009). Increasing mental health awareness and appropriate service use in older Chinese Americans: A pilot intervention. *Patient Education and Counseling, 76*(1), 143-146. |
| 8 | Walters, P., Fisher, J., & Tylee, A. (2007). Do mail-shots improve access to primary care for young men with depression? *The European Journal of Psychiatry, 21*(1), 49-54. |
| 9 | Shandley, K., Austin, D., Klein, B., & Kyrios, M. (2010). An evaluation of 'Reach Out Central': An online gaming program for supporting the mental health of young people. Health Education Research, 25(4), 563-574. |
| 10 | Berridge, B. J., Hall, K., Dillon, P., Hides, L., & Lubman, D. I. (2011). MAKINGtheLINK: A school-based health promotion programme to increase help-seeking for cannabis and mental health issues among adolescents. Early Intervention in Psychiatry, 5(1), 81-88. |
| 11 | Yau, S. S., Pun, K. H., & Tang, J. P. (2011). Outcome study of school programmes for reducing stigma and promoting mental health. *Journal of Youth Studies, 14*(1; 27), 30-40. |

*Controlled trial (n = 9)*

| 1 | Battaglia, J., Coverdale, J. H., & Bushong, C. P. (1990). Evaluation of a Mental Illness Awareness Week program in public schools. *Am J Psychiatry, 147*(3), 324-329. |
| --- | --- |
| 2 | Deane, F. P., Wilson, C. J., & Russell, N. (2007). Brief report: impact of classroom presentations about health and help-seeking on rural Australian adolescents' intentions to consult health care professionals. *J Adolesc, 30*(4), 695-699. |
| 3 | Esters, I., Cooker, P., & Ittenbach, R. (1998). Effects of a unit of instruction in mental health on rural adolescents' conceptions of mental illness and attitudes about seeking help. *Adolescence, 33*(130), 469-476. |
| 4 | Gelso, C. J., & Mckenzie, J. D. (1973). Effect of Information on Students Perceptions of Counseling and Their Willingness to Seek Help. *Journal of Counseling Psychology, 20*(5), 406-411. |
| 5 | Gould, M., Greenberg, N., & Hetherton, J. (2007). Stigma and the military: Evaluation of a PTSD psychoeducational program. *Journal of Traumatic Stress, 20*(4), 505-515. |
| 6 | Rickwood, D., Cavanagh, S., Curtis, L., & Sakrouge, R. (2004). Educating young people about mental health and mental illness: evaluating a school-based programme. *International Journal of Mental Health Promotion, 6*(4), 23-32. |
| 7 | Santor, D. A., Poulin, C., LeBlanc, J. C., & Kusumakar, V. (2007). Facilitating help seeking behaviour and referrals for mental health difficulties in school aged boys and girls: A school-based intervention. *Journal of Youth and Adolescence, 36*, 741-752. |
| 8 | Younes, N., Hardy-Bayle, M. C., Falissard, B., Kovess, V., & Gasquet, I. (2008). Impact of shared mental health care in the general population on subjects' perceptions of mental health care and on mental health status. *Soc Psychiatry Psychiatr Epidemiol, 43*(2), 113-120. |
| 9 | Wright, A., McGorry, P. D., Harris, M. G., Jorm, A. F., & Pennell, K. (2006). Development and evaluation of a youth mental health community awareness campaign - The Compass Strategy. *BMC Public Health, 6*, 215. |

*Randomised trial (n = 5)*

| 1 | Fauteux, D. J., McKelvie, S. J., & de Man, A. F. (2008). Effects of exposure to public figures' use of psychologists on attitude towards help-seeking behavior. *North American Journal of Psychology, 10*(2), 385-396. |
| --- | --- |
| 2 | Gonzalez, J. M., Tinsley, H. E. A., & Kreuder, K. R. (2002). Effects of psychoeducational interventions on opinions of mental illness, attitudes toward help seeking, and expectations about psychotherapy in college students. *Journal of College Student Development, 43*(1), 51-63. |
| 3 | Hammer, J. H., & Vogel, D. L. (2010). Men's help seeking for depression: The efficacy of a male-sensitive brochure about counseling. *The Counseling Psychologist, 38*(2), 296-313. |
| 4 | Jorm, A. F., Griffiths, K. M., Christensen, H., Korten, A. E., Parslow, R. A., & Rodgers, B. (2003). Providing information about the effectiveness of treatment options to depressed people in the community: a randomized controlled trial of effects on mental health literacy, help-seeking and symptoms. *Psychol Med, 33*(6), 1071-1079. |
| 5 | Rochlen, A. B., McKelley, R. A., & Pituch, K. A. (2006). A preliminary examination of the "Real Men. Real Depression" campaign. *Psychology of Men & Masculinity, 7*(1), 1-13. |

**Criterion 2. Targeting other disorders or problems (*n* = 8)**

*Substance use (n = 3)*

| 1 | Bennett, J. B., & Lehman, W. E. (2001). Workplace substance abuse prevention and help seeking: comparing team-oriented and informational training. *J Occup Health Psychol, 6*(3), 243-254. |
| --- | --- |
| 2 | Fox, J. C., Blank, M., Berman, J., & Rovnyak, V. G. (1999). Mental disorders and help seeking in a rural impoverished population. *International Journal of Psychiatry in Medicine, 29*(2), 181-195. |
| 3 | Grothues, J. M., Bischof, G., Reinhardt, S., Meyer, C., John, U., & Rumpf, H.-J. (2008). Differences in help seeking rates after brief intervention for alcohol use disorders in general practice patients with and without comorbid anxiety or depressive disorders. *International Journal of Methods in Psychiatric Research, 17*(Suppl1), S74-S77. |

*Suicide prevention (n = 2)*

| 1 | Klimes-Dougan, B., Yuan, C., Lee, S., & Houri, A. K. (2009). Suicide prevention with adolescents: considering potential benefits and untoward effects of public service announcements. *Crisis, 30*(3), 128-135. |
| --- | --- |
| 2 | Wyman, P. A., Brown, C. H., LoMurray, M., Schmeelk-Cone, K., Petrova, M., Yu, Q., et al. (2010). An outcome evaluation of the Sources of Strength suicide prevention program delivered by adolescent peer leaders in high schools. American Journal of Public Health, 100(9), 1653-1661. |

*Occupational trauma risk management (n = 2)*

| 1 | Bian, Y., Xiong, H., Zhang, L., Tang, T., Liu, Z., Xu, R., et al. (2011). Change in coping strategies following intensive intervention for special-service military personnel as civil emergency responders. Journal of occupational health, 53(1), 36-44. |
| --- | --- |
| 2 | Greenberg, N., Langston, V., Everitt, B., Iversen, A., Fear, N. T., Jones, N., et al. (2010). A cluster randomized controlled trial to determine the efficacy of Trauma Risk Management (TRiM) in a military population. Journal of Traumatic Stress, 23(4), 430-436. |

*Eating disorders (n = 1)*

| 1 | Becker, A. E., Franko, D. L., Nussbaum, K., & Herzog, D. B. (2004). Secondary Prevention for Eating Disorders: The impact of education, screening, and referral in a college-based screening program. *International Journal of Eating Disorders, 36*(2), 157-162. |
| --- | --- |

**Criterion 3. No extractable data (*n* = 5)**

| 1 | Biegel, D. E. (1984). Help seeking and receiving in urban ethnic neighborhoods: Strategies for empowerment. *Prevention in Human Services, 3*(2-3), 119-143. |
| --- | --- |
| 2 | Christensen, H., Griffiths, K. M., Mackinnon, A. J., Kalia, K., Batterham, P. J., Kenardy, J. (2010). Protocol for a randomised controlled trial investigating the effectiveness of an online e health application for the prevention of Generalised Anxiety Disorder. *BMC Psychiatry, 10*, 25. |
| 3 | Griffiths, K. M., Crisp, D., Christensen, H., Mackinnon, A. J., & Bennett, K. (2010). The ANU WellBeing study: a protocol for a quasi-factorial randomised controlled trial of the effectiveness of an Internet support group and an automated Internet intervention for depression. *BMC Psychiatry, 10*, 20. |
| 4 | Rees, T. (1998). N.Y. ad shop creates campaign to destigmatize mental illness. *Profiles Healthc Mark, 14*(4), 1, 4-6. |
| 5 | Robinson, J., Hetrick, S., Gook, S., Cosgrave, E., Yuen, H. P., McGorry, P. (2009). Study protocol: the development of a randomised controlled trial testing a postcard intervention designed to reduce suicide risk among young help-seekers. *BMC Psychiatry, 9*, 59. |

**Criterion 4. Not an intervention (*n* = 5)**

| 1 | Bell, R. A., Paterniti, D. A., Azari, R., Duberstein, P. R., Epstein, R. M., Rochlen, A. B. (2010). Encouraging patients with depressive symptoms to seek care: A mixed methods approach to message development. *Patient Education and Counseling, 78*(2), 198-205. |
| --- | --- |
| 2 | Frojd, S., Marttunen, M., Pelkonen, M., von der Pahlen, B., & Kaltiala-Heino, R. (2007). Adult and peer involvement in help-seeking for depression in adolescent population: A two-year follow-up in Finland. *Social Psychiatry and Psychiatric Epidemiology, 42*(12), 945-952. |
| 3 | Lorian, C. N., & Grisham, J. R. (2011). Clinical implications of risk aversion: An online study of risk-avoidance and treatment utilization in pathological anxiety. Journal of Anxiety Disorders, 25(6), 840-848. |
| 4 | Spendelow, J. S., & Jose, P. E. (2010). Does the optimism bias affect help-seeking intentions for depressive symptoms in young people? The Journal of general psychology, 137(2), 190-209. |
| 5 | Ryan, M. L., Shochet, I. M., & Stallman, H. M. (2010). Universal online interventions might engage psychologically distressed university students who are unlikely to seek formal help. *Advances in Mental Health, 9*(1), 73-83. |

**Criterion 5. Did not aim to increase help-seeking (*n* = 4)**

| 1 | Beeber, L. S., & Charlie, M. L. (1998). Depressive symptom reversal for women in a primary care setting: A pilot study. *Archives of Psychiatric Nursing, 12*(5), 247-254. |
| --- | --- |
| 2 | Buller, R., Winter, P., Amering, M., Katschnig, H., Lavori, P. W., Deltito, J. A. (1992). Center differences and cross-national invariance in help-seeking for panic disorder. A report from the cross-national collaborative panic study. *Soc Psychiatry Psychiatr Epidemiol, 27*(3), 135-141. |
| 3 | Dickinson, P., Coggan, C., & Bennett, S. (2003). TRAVELLERS: A school-based early intervention programme helping young people manage and process change, loss and transition. Pilot phase findings. *Australian and New Zealand Journal of Psychiatry, 37*(3), 299-306. |
| 4 | Wong, M. D., Andersen, R., Sherbourne, C. D., Hays, R. D., & Shapiro, M. F. (2001). Effects of cost sharing on care seeking and health status: results from the Medical Outcomes Study. *Am J Public Health, 91*(11), 1889-1894. |
